# Alcohol consumption and dependence is linked to the extent that people experience need satisfaction while drinking alcohol in two Aboriginal and Torres Strait Islander communities

**DOI:** 10.1186/s13722-021-00231-z

**Published:** 2021-04-13

**Authors:** James H. Conigrave, Emma L. Bradshaw, Katherine M. Conigrave, Richard M. Ryan, Scott Wilson, Jimmy Perry, Michael F. Doyle, K. S. Kylie Lee

**Affiliations:** 1 Faculty of Medicine and Health, Central Clinical School, NHMRC Centre of Research Excellence in Indigenous Health and Alcohol, The University of Sydney, Drug Health Services, Level 6 King George V Building, 83-117 Missenden Road, Camperdown, 2050 NSW Australia; 2 The Edith Collins Centre (Translational Research in Alcohol, Drugs and Toxicology), Drug Health Services, Sydney Local Health District, Sydney, NSW Australia; 3 Institute for Positive Psychology and Education, Australian Catholic University, Sydney, Australia; 4 Drug Health Services, Royal Prince Alfred Hospital, Camperdown, NSW Australia; 5 Aboriginal Drug & Alcohol Council SA, Aboriginal Corporation, Underdale, SA Australia; 6 National Drug Research Institute, Faculty of Health Sciences,, Curtin University, Perth, Australia; 7 Centre for Alcohol Policy Research, La Trobe University, Melbourne, VIC Australia

**Keywords:** Self-determination theory, Alcohol consumption, Basic psychological needs, Grog Survey App, Indigenous Australians

## Abstract

**Background:**

Unhealthy alcohol use is a key concern for Aboriginal and Torres Strait Islander (‘Indigenous Australian’) communities. Due to systematic disadvantage and inter-generational trauma, Indigenous Australians may be less likely to have satisfied basic psychological needs (autonomy, competence, and relatedness). When people are need-thwarted, they may engage in compensatory behaviours to feel better in the short-term. We explore the relationship between perceived basic psychological needs satisfaction and alcohol consumption use among Indigenous Australians. Better understanding the functions that alcohol may play for some Indigenous Australian drinkers may aid communities, clinicians, and policy makers in improving programs for reducing drinking-related harms.

**Methods:**

We performed a cross-sectional survey of Indigenous Australians (aged 16 years or older) living in two South Australian communities. Participants were eligible if they had consumed any alcohol in the past 12 months. Spearman correlations and linear regressions were used to determine if feeling more autonomous, competent, and related to others (need satisfied) while drinking, was linked to alcohol consumption and dependence.

**Results:**

Controlling for participant demographics, reporting feeling need satisfied while drinking was linked to drinking more alcohol per day, reporting more frequent symptoms of alcohol dependence, spending more money on alcohol, and scoring higher on the AUDIT-C.

**Conclusions:**

Unhealthy drinking may partly stem from attempts to satisfy basic psychological needs. Programs which support Indigenous Australians to meet basic psychological needs could reduce attempts to meet psychological needs through alcohol consumption.

## Introduction

Alcohol is a major cause of mortality and disease worldwide [1] and can be particularly damaging to Indigenous peoples who have been colonised [2, 3]. In Australia, Aboriginal and Torres Strait Islander (‘Indigenous Australian’) communities have identified risky drinking as a concern [4, 5]. While Indigenous peoples, worldwide, are not more likely to be current drinkers, those who drink are more likely to do so at risky levels [6]. But, the prevalence of risky drinking varies greatly within and between Indigenous Australian communities [7]. What drives this variability? Differences in experienced racism [8] and trauma [9] are likely linked to drinking risk, but individual motivations for drinking may also be important. Research using non-indigenous samples reveals that people are motivated to drink alcohol, in-part, to attain valued outcomes [10, 11]. Understanding the various functions that alcohol has for Indigenous Australians may help clinicians and policy makers develop tailored interventions to reduce alcohol-related harms.

### Indigenous Australians and basic psychological needs

*Self-determination theory* (SDT) is a macro-theory of human motivation and well-being [12]. SDT posits that in order to thrive, people need to feel autonomous (free and self-determined), competent (able to be effective), and related (connected to others) [13]. When these needs are satisfied, people experience improved well-being and health [12, 14, 15]. Conversely, frustration of basic psychological needs leads to ill-being and distress [12].

In addition to supporting well-being, basic psychological need satisfaction serves to motivate many behaviours [12]. Boosting basic psychological need satisfaction has been found to motivate physical training [16], healthier eating [14, 17], better school engagement [18], and a host of other beneficial outcomes [12]. However, when people are otherwise need frustrated, they may become motivated to engage in harmful behaviours to satisfy their needs in the short-term [19]. For instance, research has shown that people experiencing basic psychological need frustration are more likely to become addicted to need-satisfying video games [20–24]. These need-satisfying compensatory behaviours may extend beyond video games. Alcohol consumption could be used by some people to compensate for unmet basic psychological needs.

The role of alcohol consumption in compensating for frustrated basic psychological needs may be especially relevant for Indigenous populations that have been colonised. Indeed, self-determination theory may be a useful lens through which to better understand Indigenous well-being in general [25]. Western colonisation has dispossessed Indigenous peoples of their lands, and forced them into systems which, at times, have not valued their contributions [2, 3]. Indigenous Australians can be marginalised from mainstream society and face discrimination [26], socio-economic disadvantage [27], and poorer health [27] relative to other Australians. Thus, due to societal injustice and a lack of opportunity, many Indigenous Australians may have unsatisfied basic psychological needs [28].

### Drinking to compensate for thwarted needs

Work with non-indigenous populations has found people are motivated to drink, not only to cope with distress (coping motives) [29], but also to feel good (enhancement motives) [30, 31], and to regulate social functioning (to gain social rewards, and to conform to others) [32–35]. By regulating mood, and connection to others, alcohol may be used by some Indigenous Australians to meet basic psychological needs. That is, they may drink, in part, to feel more autonomous, competent, and connected to others. However, no study to date has examined whether risky drinking is linked to the extent that alcohol is perceived to satisfy basic psychological needs. If Indigenous Australians are drinking to meet needs, then risky drinking should be considered in the context of broader individual well-being.

In the current paper, we aimed to determine whether Indigenous Australians experience need satisfaction while drinking; and whether the extent to which Indigenous Australians find alcohol need-satisfying is linked to greater alcohol consumption and dependence. Drinking may satisfy the need for autonomy by making users feel disinhibited, free, and providing them with choices (what to drink, where, and with whom). Disinhibition may also help users feel connected to others (relatedness) [12, 36], especially if they are socially anxious or cannot freely talk to others when sober [35]. Finally, alcohol can temporarily enhance self-worth and confidence thus satisfying the need of competence [33]. Taken together, we hypothesise that participants who feel autonomous, connected to others, and competent while drinking, will drink more alcohol and report more frequent symptoms of alcohol dependence.

## Methods

This study is part of a broader project to describe self-reported alcohol use behaviours in Indigenous Australians [37].

### Research ethics

Ethical approval was obtained from the Aboriginal Health Council SA (AHCSA; Ref: 04/15/621) and from Metro South Health Human Research Ethics Committee in Queensland (Ref: HREC/16/QPAH/293).

#### Indigenous involvement

Australian ethical guidelines stress that Indigenous Australians should be collaborators on, and benefit from, research that draws upon their communities’ resources [38]. Study methods were designed by investigators in consultation with the Aboriginal Drug and Alcohol Council of South Australia (ADAC). Three study authors are Indigenous Australian.

### Participants

Participants were Indigenous Australians, aged 16 years or older, who had consumed alcohol in the past year. They were recruited from two Indigenous Australian communities in South Australia (one urban and one remote). The urban sample was stratified to match local demographics [37]. The remote sample aimed to capture all local community members documented in the 2016 Australian Census of Population and Housing [39].

### Materials

All data were collected using ‘The Grog Survey App’ (grog is a local colloquialism for alcohol) [40]. The Grog App was developed to collect data on alcohol consumption in a culturally acceptable way for Indigenous participants [41, 42]. Participants were given headphones to listen to questions in English or Pitjantjatjara (a local Indigenous Australian language). Translations were back-translated into English to verify their accuracy [43]. The App was self-administered with support from research assistants (who included Indigenous Australian health professionals).

#### Alcohol satisfaction of psychological needs

Basic psychological needs satisfaction and frustration scales [44] have been translated into multiple languages and adapted to many domains including: education [45], physical education [46], sport [47], romantic relationships [48], and the workplace [49]. We adapted items from the ‘Basic Psychological Need Satisfaction in Relationships’ scale [48], by replacing references to partners with references to alcohol consumption. For example the item: “When I am with my partner, I feel free to be who I am” became “When I drink, I feel free to be who I am.” Language in the scale was adapted by Indigenous Australian researchers, local language speakers, study investigators, and health professionals to ensure suitability for Indigenous Australians, and that the meaning of items was retained. We refer to this modified scale as the “Alcohol Satisfaction of Psychological Needs” (ASPN) scale. The nine items are: “When I drink, I feel free to be who I am,” “When I drink I feel like I have a say in what happens, like I can voice my opinions,” “I have to drink because friends or family want me to,” “When I drink I feel more confident,” “When I drink I feel like I’m good at things in general,” “Drinking makes me feel like I can’t do things very well,” “Drinking makes me feel close to others,” “Drinking makes me feel like part of the group” and, “When I drink, I feel lonely (even if others are around).” Items were scored on a six point Likert scale ranging from strongly disagree to strongly agree.

#### AUDIT-C

The Alcohol Use Disorders Identification Test: Consumption questions (AUDIT-C) is a three-item screening tool used to identify individuals at risk from drinking [50–52]. The AUDIT-C is widely used in Indigenous Australian contexts [53, 54]. Questions were adapted by study investigators for an Indigenous Australian audience: “Some people drink grog most days, while others drink ‘once in a blue moon.’ How often have you had any grog in the last 12 months?” Responses were on a five-point scale: “never,” “once in a blue moon (less than once a month),” “sometimes (2–4 times a month),” “2–3 times a week,” “Most days or every day.” The second item was: “How much grog do you have on a typical day when you drink?” Responses were on a five-point visual scale presented in participants’ preferred type of alcohol (beer, cider, wine, spirits, or port/sherry). The response scale depicted 1–2, 3–4, 5–6, 6–9 and 10+ standard drinks (each 10 g of ethanol). The final item was paired with a visualisation of six standard drinks, shown in participants preferred alcohol type: “How often would you drink this much grog or more in 1 day (24 h)?” Responses were on the same five-point scale as for the first question ranging from “Never” to “Most days or every day.”

#### Alcohol consumption

The Finnish method [40, 55] was used to estimate drinking intensity (based on the last two drinking occasions) and frequency (based on the last four drinking occasions). The Finnish method has been found to be a valid and acceptable tool for measuring the alcohol consumption of Indigenous Australians [40, 41]. Drinking intensity (amount consumed per drinking occasion) was calculated by averaging the standard drinks (10 g of ethanol) of alcohol consumed over the last two occasions (in the past 12 months). Drinking occasion frequency was calculated by dividing the total number of drinking occasions by the total duration (in days) over which they were consumed [40]. An estimate of average standard drinks consumed per day was calculated by multiplying the average standard drinks per drinking occasion by the number of drinking occasions per day [40].

#### Alcohol dependence

Dependence questions were operationalised from ICD-11 diagnostic guidelines and worded for an Indigenous Australian audience [56]. The first question was: “Some people feel like grog is the boss of them. How often do you feel grog makes all the decisions? (so you could not stop drinking, even if you tried).” The second question was “Some people’s hands shake when they stop drinking or before their first drink of the day. In the last 12 months, how often does this happen to you?” The third question was: “Some people spend more time drinking than doing other things they need to do, like looking after family, culture or work. In the last 12 months, how often does this happen to you?” Responses were given on a five-point scale ranging from “never” to “most days or every day.” A total dependence score was calculated by summing scores across the three items.

#### Money spent on alcohol

Participants were asked: “On a day when you drink, how much money do you spend on grog?” Participants answered on a five-point scale: ‘$0–25,’ ‘$26–50,’ ‘$51–75,’ ‘$76–99,’ ‘$100 or more.’

### Data analysis

All analyses were performed using R version 4.0.4 (2021-02-15) [57]. Responses to the ASPN were visualised for each item using the sjPlot library [58]. We performed exploratory factor analysis (EFA) [59] to determine the factor structure of the ASPN. The number of factors was selected using principal component analysis and multi-dimensional scaling as implemented in the ‘stats’ [57] and ‘corx’ [60] packages, respectively. Multi-dimensional scaling visualises item similarity. It achieves this by converting inter-item correlations to distances and then mapping items to an abstract two-dimensional space—items closer together are more similar [61]. EFA [59] was performed with the ‘psych’ [62] package. Factor scores were extracted from the EFA to be used in subsequent analyses. Spearman correlations were used to assess the link between ASPN and consumption and dependence. Multivariate linear regressions were performed using the 'stats' library [57] to determine the links between the basic psychological needs factors and alcohol consumption and dependence controlling for participant age, gender, and remoteness.

## Results

### Participant characteristics

We approached 799 people to take the Grog Survey App. In 24 cases (3.00%) the App was not completed. This was due to participants stopping early (n = 2), technical problems (n = 4), or because the participant was uncomfortable and did not wish to proceed (n = 8). Of those who completed the survey, one in five (22.97%) had not consumed alcohol in the past 12 months and were excluded from further analysis. The final sample of current drinkers included 597 participants. The average age of participants was 36.14 ($SD$ = 14.74). Just over half of participants were female (n = 300; 50.25%).

### Need satisfaction from consuming alcohol

**Fig. 1:**
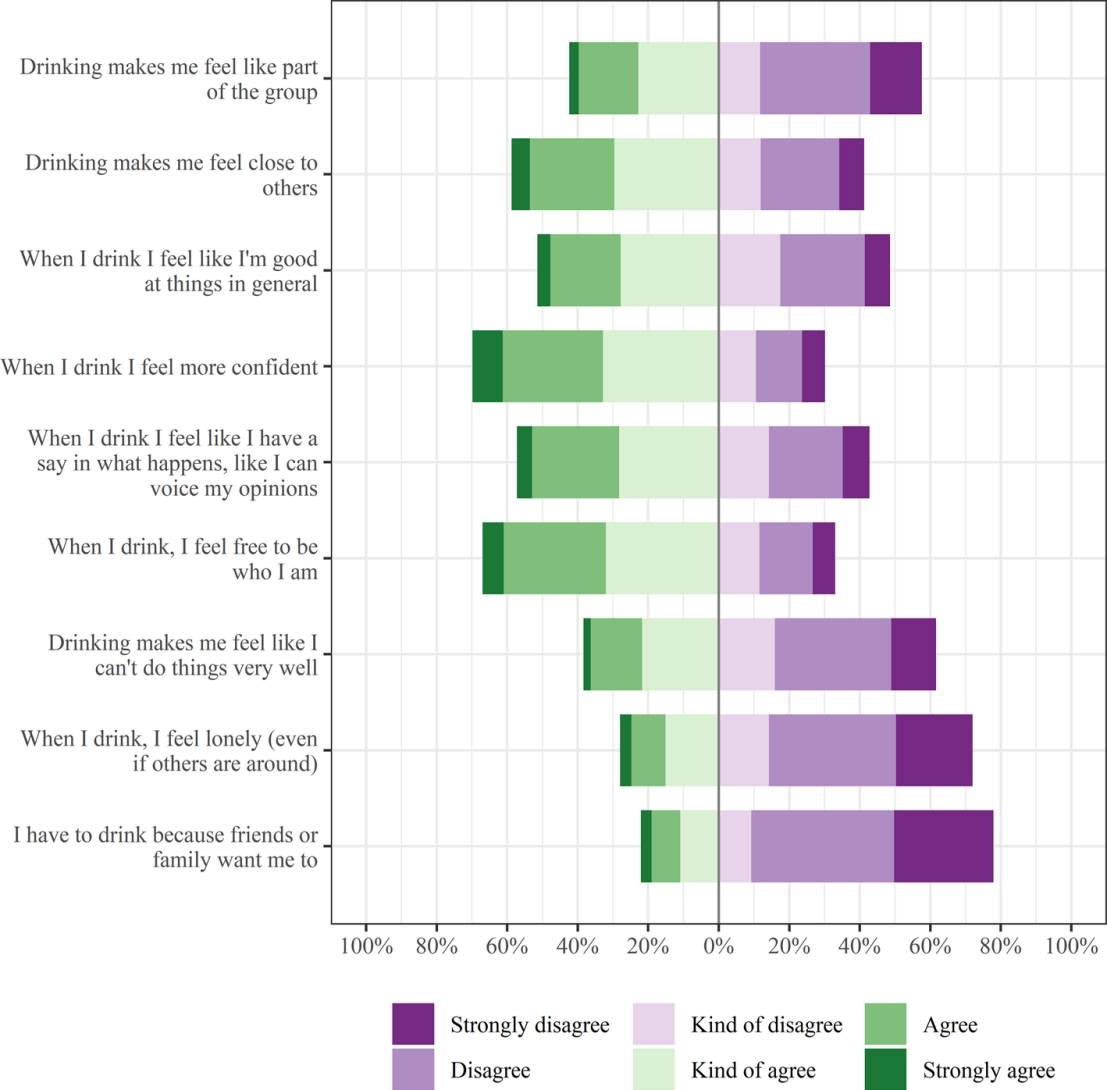
Responses to the alcohol satisfaction of psychological needs questions. The x-axis displays the percentage of respondents. The area to the right of the central line displays the percentage of participants who disagreed with a given question. The area to the left shows the percentage of those who agreed

Figure 1 shows how participants responded to each question about how need satisfied they feel while drinking. There was large variability in responses to questions about whether alcohol satisfied needs. Respondents tended to agree with statements about alcohol supporting needs, and disagreed with statements about alcohol thwarting needs.

### Inter-correlation matrix (ASPN)

Descriptive statistics and Pearson correlations for the ASPN items are presented in Table 1. Most items tended to have flat distributions (relative to the normal distribution; kurtosis < 3) meaning that there was large variability in responding across each item. There were moderate to strong correlations across the various needs (autonomy, competence, and relatedness). For instance, feeling like belonging to the group while drinking (relatedness satisfaction) was positively correlated with feeling pressure to drink (autonomy thwarting; ${r}_{s}$ = 0.58, $p$ < 0.001). Feeling closer to others (relatedness satisfaction) while drinking was strongly correlated with feeling competent (competence satisfaction; ${r}_{s}$ = 0.63, $p$ < 0.001). The three items about alcohol thwarting needs were positively correlated (albeit in most cases weakly) with items about alcohol supporting needs. This may reflect that people experience both need satisfaction and frustration while drinking.

**Table 1:** Descriptive statistics and Pearson intercorrelations: alcohol satisfaction of psychological needs (ASPN) items

	1	2	3	4	5	6	7	8	$\mathrm{M}$	$\mathrm{SD}$	$\mathrm{Skew}$	$\mathrm{Kurtosis}$
1. Belonging	–								3.04	1.43	0.19	1.83
2. Feel closer	0.47***	–							3.57	1.38	− 0.23	2.00
3. Feel competent	0.43***	0.63***	–						3.40	1.33	− 0.07	2.02
4. Feel confident	0.23***	0.36***	0.42***	–					3.89	1.35	− 0.55	2.51
5. Can voice opinions	0.35***	0.47***	0.54***	0.36***	–				3.55	1.37	− 0.24	2.02
6. Feel free	0.35***	0.46***	0.52***	0.53***	0.54***	–			3.80	1.34	− 0.51	2.38
7. Feel useless	0.31***	0.25***	0.22***	0.17***	0.27***	0.21***	–		2.98	1.35	0.27	1.99
8. Feel alone	0.29***	0.21***	0.24***	0.17***	0.18***	0.21***	0.40***	–	2.64	1.39	0.67	2.46
9. Feel pressured	0.58***	0.25***	0.29***	0.07	0.20***	0.21***	0.30***	0.33***	2.40	1.37	0.99	3.04

*p < 0.05; **p < 0.01; ***p < 0.001; $n$ = 597 Descriptive statistics are for items score (range 1–6); Skew = skewness (distribution asymmetry); Kurtosis = distribution flatness, the normal distribution has a kurtosis value of 3

### Factor analysis

#### Determining the number of factors

**Fig. 2:**
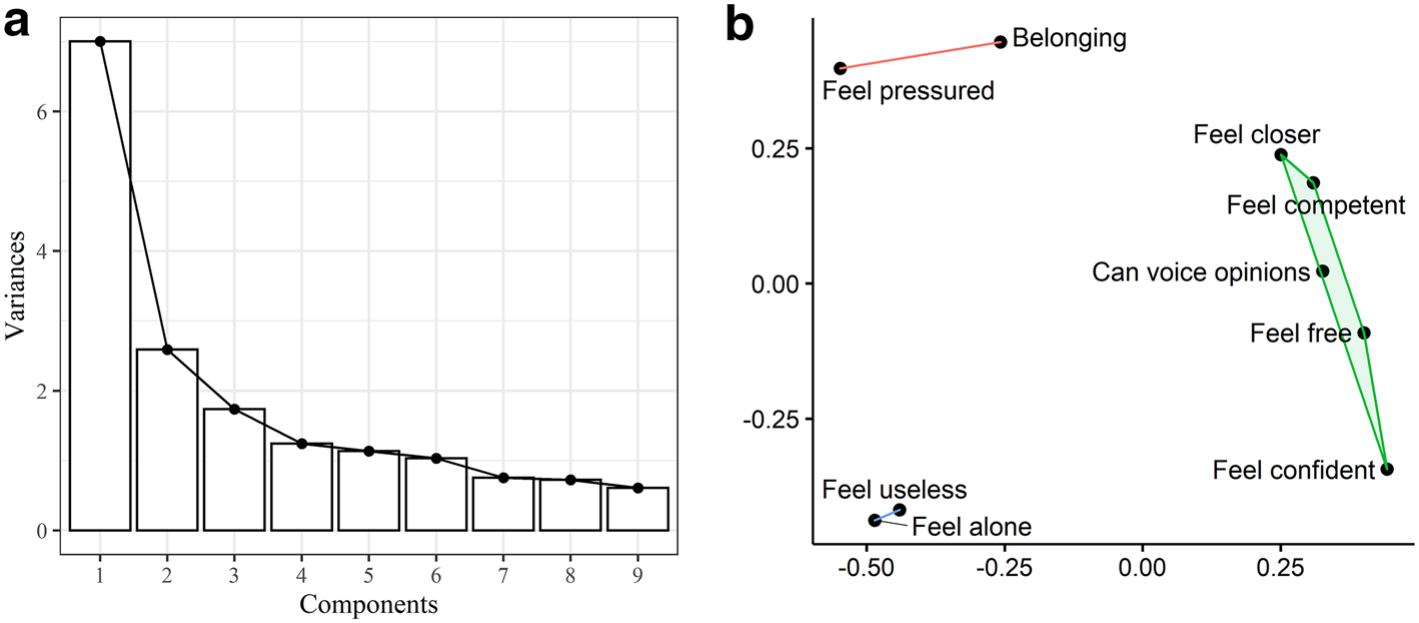
Scree plot (**a**) and Multi-Dimensional Scaling (MDS) of items (**b**). The scree plot describes the variance explained by each principal component. The multi-dimensional scaling plot visualises similarity between items as distances. Items closer to each other are more similar (correlated)

To determine how the items clustered together, we performed factor analysis. Principal component analysis was used to identify the optimal number of factors. The cumulative proportion of variance explained by each factor is presented in Fig. 2a. The first three principal components explained 67.33% of the total variance. A clear point was not observed where adding additional components added trivial amounts of variance. To aid the selection of the number of factors, multi-dimensional scaling was performed (using two dimensions) and graphed (Fig. 2b). Three major clusters of items were apparent from visual inspection of the distance between items. Feeling alone and useless clustered together, as did feeling pressure and belonging while drinking. The remaining items all referenced feeling need satisfied while drinking and formed their own larger cluster. To obtain factor loadings, we performed an EFA specifying three factors.

#### Exploratory factor analysis

Maximum likelihood exploratory factor analysis was performed with varimax rotation for three factors (Table 2). The first factor (‘Need satisfaction’) explained 52.26% of the total variance. This factor was comprised of all but one need satisfaction item: ‘Feeling closer,’ ‘Feeling competent,’ ‘Feeling confident,’ ‘Can voice opinions’ and ‘Feel Free.’ The second factor (‘Conformity’) explained 27.67% of variance. The items which loaded most strongly on this factor were ‘Belonging,’ and ‘Feel pressured.’ The third factor (‘Social exclusion’) accounted for 20.07% of variance. The items which loaded most strongly on this factor were both need frustration items: ‘Feel alone’ and ‘Feel useless.’

**Table 2:** Alcohol satisfaction of psychological needs (ASPN) factor loadings

Item	Factor 1	Factor 2	Factor 3
Belong	0.32	0.72	0.21
Feel closer	0.65	0.33	0.08
Feel alone	0.14	0.18	0.60
Feel competent	0.72	0.29	0.10
Feel confident	0.60	− 0.03	0.15
Feel useless	0.17	0.19	0.55
Can voice opinions	0.65	0.16	0.16
Feel free	0.70	0.11	0.18
Feel pressured	0.07	0.67	0.32

Factor 1 = Need satisfaction; Factor 2 = Social pressure; Factor 3 = Social exclusion. Item content is abbreviated, see methods for item wording

### Relationships between ASPN factors and outcome variables

ASPN factor scores were extracted for each participant, and correlated with indices of alcohol consumption and dependence. As metrics of alcohol consumption are skewed, Spearman correlations were computed (Table 3). All three factors were positively correlated with standard drinks consumed per day, money spent on alcohol, AUDIT-C score, and symptoms of alcohol dependence. Feeling more excluded (feeling alone and useless) while drinking was strongly linked to more frequent symptoms of dependence. Alcohol consumption (AUDIT-C score, standard drinks consumed per day and money spent on alcohol) was mostly strongly linked to feeling need-satisfied while drinking.

**Table 3:** Spearman correlations between the factor scores and indicators of drinking

	1	2	3	4	5	6	$\mathrm{Median}$	$\mathrm{IQR}$
1. Need satisfaction	–						0.08	1.21
2. Conformity	0.14***	–					− 0.16	1.23
3. Exclusion	0.11**	0.24***	–				− 0.11	0.99
4. Standard drinks/day	0.23***	0.12**	0.11**	–			0.29	1.17
5. Dependence	0.22***	0.28***	0.40***	0.35***	–		0.00	3.00
6. AUDIT-C score	0.24***	0.14***	0.12**	0.70***	0.40***	–	5.00	4.00
7. Money spent on alcohol	0.25***	0.20***	0.12**	0.48***	0.27***	0.50***	2.00	2.00

*p < 0.05; **p < 0.01; ***p < 0.001; $n$ = 597; all correlations are Spearman rho. Items 1–3 are the three identified factors from the ASPN scale. Standard drinks / day reflects the average amount of alcohol consumed on any day of the year. Money spent on alcohol, the AUDIT-C and Dependence are ordinal scales

### Multivariate regressions

The three ASPN factors (need satisfaction, conformity, and exclusion) were included in multivariate linear regressions (OLS) which predicted the four different outcomes: standard drinks per day, dependence symptom frequency, money spent on alcohol, and AUDIT-C score (Tables 4 and 5). Each model controlled for age, gender, and remoteness. Preliminary analyses demonstrated that the model predicting standard drinks per day resulted in biased residuals. To ensure regression assumptions were met, we log-transformed the dependent variable (standard drinks per day) and re-fit this model.

**Table 4:** Multivariate regressions predicting standard drinks per day and dependence score with basic psychological needs from alcohol

Predictor	Coefficient [95% CI]	SE	$t$	$p$
Log standard drinks/day^a^				
Intercept	− 1.38 [− 1.82, − 0.95]	0.22	− 6.19	< 0.001*
Age	− 0.01 [− 0.02, 0.00]	0.01	− 2.32	0.021*
Male	1.14 [0.84, 1.44]	0.15	7.53	< 0.001*
Remote	0.29 [− 0.29, 0.87]	0.30	0.99	0.32
Need satisfaction^†^	0.48 [0.33, 0.63]	0.08	6.17	< 0.001*
Conformity^†^	0.15 [− 0.01, 0.31]	0.08	1.88	0.061
Exclusion^†^	0.14 [− 0.01, 0.29]	0.08	1.79	0.074
Dependence^†^				
Intercept	− 0.29 [− 0.50, − 0.08]	0.11	− 2.72	0.007*
Age	0.00 [0.00, 0.01]	0.00	1.95	0.052
Male	0.20 [0.06, 0.35]	0.07	2.81	0.005*
Remote	0.16 [− 0.11, 0.44]	0.14	1.16	0.25
Need satisfaction^†^	0.13 [0.06, 0.21]	0.04	3.55	< 0.001*
Conformity^†^	0.17 [0.10, 0.25]	0.04	4.51	< 0.001*
Exclusion^†^	0.34 [0.26, 0.41]	0.04	8.98	< 0.001*

*p < 0.05; ^†^standardised variable; $$n$$ = 597

^a^Standard drinks per day was log-transformed, to interpret coefficients (${\beta }_{i}$) as percent increases for log-transformed outcomes (y) the following formula can be used: $\Delta{\%}y=({e}^{{\beta }_{i}}-1)*100$. Dependence items were summed to create an aggregate measure (range 0–12)

**Table 5:** Multivariate regression predicting money spent on alcohol and AUDIT-C score with basic psychological needs from alcohol

Predictor	Coefficient [95% CI]	SE	$t$	$p$
Money spent on alcohol^†^				
Intercept	0.02 [− 0.21, 0.24]	0.11	0.15	0.88
Age	0.00 [− 0.01, 0.00]	0.00	− 1.66	0.098
Male	0.21 [0.05, 0.36]	0.08	2.66	0.008*
Remote	0.59 [0.29, 0.88]	0.15	3.86	< 0.001*
Need satisfaction^†^	0.18 [0.10, 0.26]	0.04	4.44	< 0.001*
Conformity^†^	0.13 [0.05, 0.21]	0.04	3.24	0.001*
Exclusion^†^	0.05 [− 0.02, 0.13]	0.04	1.35	0.18
AUDIT-C score				
Intercept	4.48 [3.87, 5.09]	0.31	14.39	< 0.001*
Age	0.00 [− 0.01, 0.02]	0.01	0.36	0.72
Male	1.21 [0.80, 1.63]	0.21	5.74	< 0.001*
Remote	0.14 [− 0.67, 0.95]	0.41	0.35	0.73
Need satisfaction^†^	0.59 [0.38, 0.80]	0.11	5.42	< 0.001*
Conformity^†^	0.18 [− 0.03, 0.40]	0.11	1.66	0.098
Exclusion^†^	0.17 [− 0.04, 0.39]	0.11	1.57	0.12

*p < 0.05; ^†^standardised variable; $$n$$ = 597

Controlling for all demographic factors, the general psychological need satisfaction factor predicted consuming more standard drinks per day, more frequent symptoms of dependence, more money spent on alcohol, and higher AUDIT-C scores. For each standard deviation increase in feeling need-satisfied while drinking alcohol, the number of standard drinks consumed per day increased by an average of 61.72% [95% CI 38.77 88.45] (Table 4). Controlling for demographics, both the conformity and exclusion factors predicted more frequent dependence symptoms (Table 4). Additionally, the conformity factor predicted spending more money on alcohol (Table 5).

## Discussion

We aimed to determine whether experiencing alcohol consumption as satisfying basic psychological needs (autonomy, competence, and relatedness) is linked to risky drinking and alcohol dependence among Indigenous Australians. We found that participants who reported feeling need-satisfied while drinking reported higher alcohol consumption, spent more money on alcohol, scored higher on the AUDIT-C, and reported more frequent symptoms of alcohol dependence. These relationships were observed even when controlling for participant demographics (age, gender and remoteness). While this study was cross-sectional (causal orderings and potential mediating factors could not be assessed) these patterns of results suggest that regulating basic psychological needs could be a motivating factor behind risky drinking for Indigenous Australians. If this is the case, then clinical and public health strategies which support basic psychological needs (that make clients feel self-determined, competent, and connected to others) would probably help reduce risky drinking. Moreover, clinicians and policy makers should be cautioned that some Indigenous Australians who are using alcohol to meet basic psychological needs, may need social, or other supports put in place such that restricting their drinking does not come at a cost to their well-being [19].

### Needs compensation and dependence

In our sample of Indigenous Australians, participants who felt needs-satisfied while drinking, consumed more alcohol and reported more frequent symptoms of dependence. Alcohol consumption could be used by some as a ‘quick fix’ to satisfy basic psychological needs [35]. Over short spans of time, consuming alcohol may enhance mood and feelings of social connection. The short-term enhancement of basic psychological needs could make alcohol particularly rewarding and encourage future use. However, if underlying circumstances causing need deficits are not resolved, alcohol consumption could become habitual and harms from alcohol—including dependence—more likely [63].

While positive feelings were associated with drinking, feeling socially excluded (alone and useless) was also associated with higher alcohol consumption and more frequent symptoms of dependence. Having negative experiences while drinking could indicate that people use alcohol to cope with distress (coping motives) [35], or may reflect self-critical emotions resulting from excessive drinking or alcohol dependence [56]. Regardless, it seems that drinking may both satisfy and thwart basic psychological needs. People at times may drink alcohol to meet psychological needs, and at other times drink despite alcohol consumption making their mood and well-being worse. While drinking in moderation may have short-term positive psychosocial effects, these are likely to diminish and become harms as consumption increases and is sustained over longer periods of time [64].

Many Indigenous Australians may find alcohol consumption need satisfying, yet do not engage in risky drinking. As with video gaming addictions, the extent to which drinking is perceived to meet needs may not be linked to increased drinking risk if basic psychological needs (in general) are being consistently satisfied [22]. The interaction between basic psychological need satisfaction from drinking and need satisfaction in general life could further shed light on Indigenous Australian drinking risk.

### Implications

The relationship between finding alcohol need satisfying and drinking, may be helpful for policy makers, communities, and clinicians to consider. If people are drinking to experience need satisfaction, restricting alcohol will also restrict need satisfaction. Thus, rather than focusing only on the removal of alcohol, community-based or clinical programs which facilitate Indigenous Australians’ need satisfaction in other areas of life may be effective in reducing risky drinking. Social activity groups may be especially helpful as they can help clients to connect with others (meeting the need for relatedness), while engaging in intrinsically motivating challenges (meeting the need for autonomy and competence). Participation in weekly social activities has been linked to less frequent alcohol use in Aboriginal Canadian youth [65]. Activity groups could include community choirs, which have been found to reduce social isolation, and improve well-being for Indigenous Australians living with chronic diseases [66]. There are also over 1000 ‘Men’s sheds’ in Australia [67]. Men’s sheds (some exclusively for Indigenous Australians) are spaces freely available to men to work together on wood-work or mechanical projects [67]. Joining a shed has been linked to increased numbers of friends, satisfaction of social needs, and a greater sense of achievement [68]. Cultural activity groups (e.g. painting, dancing, basket-weaving, or cooking) may also help participants meet basic psychological needs and build resilience [69, 70].

Aboriginal community controlled heath services have long provided holistic, culturally-informed care [71] which may support the basic psychological needs of people at risk from drinking. For example, some services run cultural activity groups to strengthen emotional health and identity [72–74]. Unfortunately, the effectiveness of community-led programs are often not evaluated due to a lack of funding. But Indigenous peoples from many countries have emphasised the importance of identity, and connection to both community and culture [71]. Additionally, many Indigenous and non-indigenous health professionals intuitively understand that risky drinking does not take place in a vacuum separate to other life stressors and strengths. The utility of the current research is that it offers a potential unifying framework for understanding the relationship between basic psychological needs satisfaction, and problematic alcohol use. This framework could help researchers in identifying mediating pathways which explain why clinical programs which support basic psychological needs also reduce risky drinking.

### Limitations

Items for the Alcohol Psychological Need Satisfaction scale (ASPN) were derived from similar scales in other domains. However, these items have not been applied to the domain of alcohol previously. The factor structure of the introduced ASPN scale did not mirror the structure of general basic psychological needs. While basic psychological needs have a stable three factor structure, different activities may satisfy needs in different ways [12]. Previous research has found that the three needs are highly correlated with each other which may explain why a general need satisfaction factor was found [44]. The other two factors (which emphasise conformity and exclusion) could highlight the social roles alcohol may be playing in Indigenous Australian communities, which are known for collectivist kinship cultures [75, 76]. To test the stability of this domain-specific factor structure, this study should be repeated with a larger battery of items. Including general basic psychological need satisfaction and frustration items in future studies would help clarify whether need satisfaction from alcohol predicts consumption in all people, or only in those who have unsatisfied needs in other domains of life. Mental health and trauma are probably important contextual factors for understanding alcohol consumption and dependence. Perhaps people with trauma or poorer mental health are especially likely to engage in risky drinking as a strategy to meet un-met basic psychological needs. Finally, no non-indigenous participants were included in this study. The inclusion of non-indigenous participants would help determine whether these findings are specific to Indigenous Australians.

## Conclusion

How Indigenous Australians feel while drinking is linked to the amount of alcohol consumed, and the frequency of symptoms of alcohol dependence. Participants who reported feeling their basic psychological needs were satisfied while drinking tended to report higher consumption and more frequent symptoms of dependence. Supporting Indigenous Australians to meet basic psychological needs in safe ways could reduce risky drinking.

## Data Availability

Data for this project is stored at the University of Sydney based at Drug Health Service, KGV Building, Missenden Road, Camperdown New South Wales, 2050 Australia.
